# Strata temperatures and geothermal resource evaluation in the Dongpu Depression, Bohai Bay Basin, North China

**DOI:** 10.1038/s41598-023-30760-3

**Published:** 2023-03-03

**Authors:** Yong Qiao, Yinhui Zuo, Shiqi Tu, Jiazhen Zhang, Meihua Yang, Tao Zhang

**Affiliations:** 1 School of Geosciences, Yangtze University, Wuhan, 430100 Hubei China; 2 State Key Laboratory of Oil and Gas Geology and Exploitation, Chengdu University of Technology, Chengdu, 610059 China; 3 Key Laboratory of Tectonics and Petroleum Resources, Ministry of Education, China University of Geosciences, Wuhan, 430074 China

**Keywords:** Geology, Solid Earth sciences

## Abstract

The development of geothermal resources in the Dongpu Depression can improve not only the economic benefits of the oilfield but also the ecological environment. Therefore, it is necessary to evaluate the geothermal resources in the region. Based on the heat flow, geothermal gradient and thermal properties, the temperatures and their distribution in different strata are calculated using geothermal methods, and the geothermal resource types of the Dongpu Depression are identified. The results show that the geothermal resources include low-temperature, medium-temperature and high-temperature geothermal resources in the Dongpu Depression. The Minghuazhen and Guantao Formations mainly include low-temperature and medium-temperature geothermal resources; the Dongying and Shahejie Formations include low-temperature, medium-temperature and high-temperature geothermal resources; the Ordovician rocks mainly include medium-temperature and high-temperature geothermal resources. The Minghuazhen, Guantao and Dongying Formations can form good geothermal reservoirs and are favorable layers for exploring low-temperature and medium-temperature geothermal resources. The geothermal reservoir of the Shahejie Formation is relatively poor, and the thermal reservoirs may be developed in the western slope zone and the central uplift. The Ordovician carbonate strata can provide thermal reservoirs for geothermal resources, and the Cenozoic bottom temperature is more than 150 °C except for most of the western gentle slope zone. In addition, for the same stratum, the geothermal temperatures in the southern Dongpu Depression are higher than those in the northern depression.

## Introduction

Geothermal is a sustainable and renewable energy resource and features an operationally reliable, stable, and environmentally friendly nature^1–5^. Its development can not only reduce carbon dioxide emissions but also improve the efficiency of oilfields^2,6^.

The oilfields have a large number of geological, geophysical and geochemical data, and have a comprehensive understanding of geological models and geothermal reservoir evaluation, which provide important support for geothermal resource evaluation and development^7,8^. Meanwhile, the existing surface facilities, wellbores and useful data empower the oilfield geothermal project with minimized risk, reduced cost and significant convenience^9,10^. Therefore, the geothermal resource evaluation and development in the oilfields has been continuously studied, and a considerable amount of geothermal reservoirs are reported in worldwide oilfields^11–13^. For instance, 3D numerical models for temperature prediction and reservoir simulation in the geothermal project Den Haag^13^, and abandoned deep hydrocarbon reservoirs and dry wells in the Croatian part of the Pannonian Basin have been considered as geothermal energy sources^11^.

The main geothermal reservoirs in the Dongpu Depression include the Minghuazhen, Guantao, and Dongying Formations and Ordovician layers, but the temperature distribution of each set of geothermal reservoirs has not been studied. Strata temperatures are of great significance for geothermal resource evaluation and are important factors in the classification and potential of geothermal resources^13^.

There is a large population and high energy consumption around the Dongpu Depression, especially from coal heating in winter, which causes serious fog and haze in this area^14^. We could use geothermal energy instead of coal for heating in winter to improve environmental quality. By using geothermal resources hotter than 80 °C, it would be possible to generate 830 × 10^8^ GJ of electrical power generation in the Dongpu Depression, equivalent to approximately 28 × 10^8^ tons of standard coal; the resources with temperatures of 60–80 °C could be directly used to supply 930 × 10^8^ GJ of heat, equivalent to approximately 32 × 10^8^ tons of standard coal; the energy that could be produced from geothermal resources with temperatures of 40–60 °C is 690 × 10^8^ GJ, equivalent to approximately 24 × 10^8^ tons of standard coal^15^.

Deep Ordovician carbonate rocks generally have a large geothermal potential in China^14^. The famous Xiongxian geothermal fields (Ordovician Wumishan Formation)^16^ and the Qingfeng and Nanle regions (Ordovician)^14^ have been providing geothermal water from carbonate reservoirs for residential heating. The Ordovician is well-developed in the Dongpu Depression, and the conditions of the geothermal reservoir, caprock and water source are good^14^. Petroleum development has already entered the middle and late production stages, and the average water cut of produced fluid in oil wells is over 90% in the Dongpu Depression^15^. There are more than 3000 oil and water wells in the oilfield and 13 joint stations^15^. Oily wastewater is separated in the amount of nearly 8.6 × 10^4^ m^3^ every day and 0.3 × 10^8^ m^3^ per year, and the average temperature of the wastewater is 43.2 °C^15^. The treatment of oilfield sewage reduces the economic benefits of the oilfield, and the treatment and discharge of sewage have caused great damage to the environment^17^.

In this paper, based on the present geothermal field, the temperature distribution characteristics of each set of strata are calculated by the geothermal method set in^18^, which provides a basis for the evaluation and development of geothermal resources in this region.

## Geological settings

**Figure 1:**
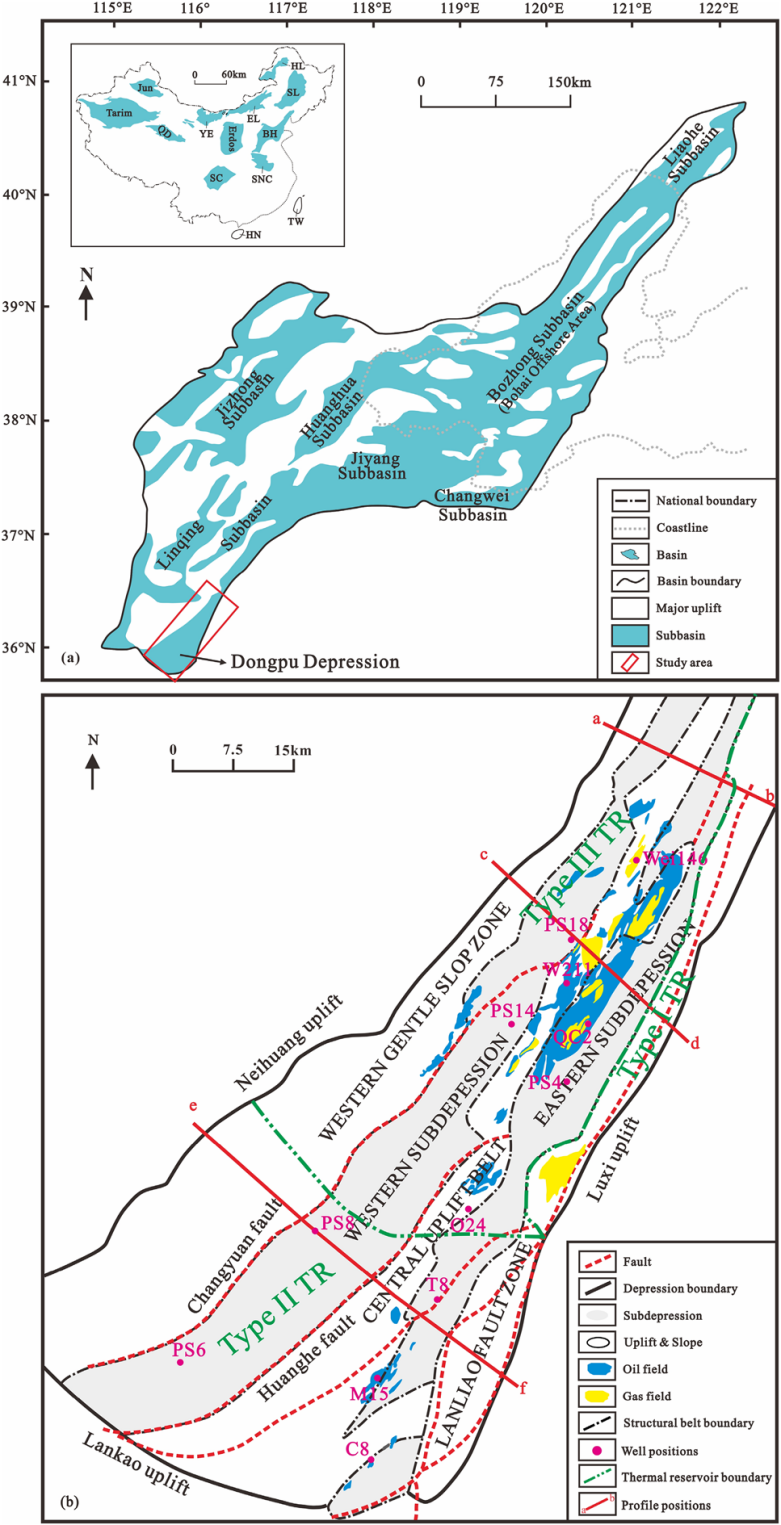
(**a**) Structural unit division of the Bohai Bay Basin^21^; (**b**) Structural unit division of the Dongpu Depression. TW = Taiwan; HN = Hainan; SC = Sichuan; BH = Bohai Bay; Jun = Junggar; QD = Qaidam; YE = Yingen-Ejinaqi; EL = Erlian; HL = Hailaer; SL = Songliao; SNC = Southern part of North China; GTR = Geothermal reservoirs; Type I GTR is a well-developed karst carbonate rock; Type II GTR is a medium developed karst carbonate rock; Type III GTR is an underdeveloped karst carbonate rock. Figures were produced by CorelDRAW Graphics Suite X8 (https://www.corel.com/en/).

**Figure 2:**
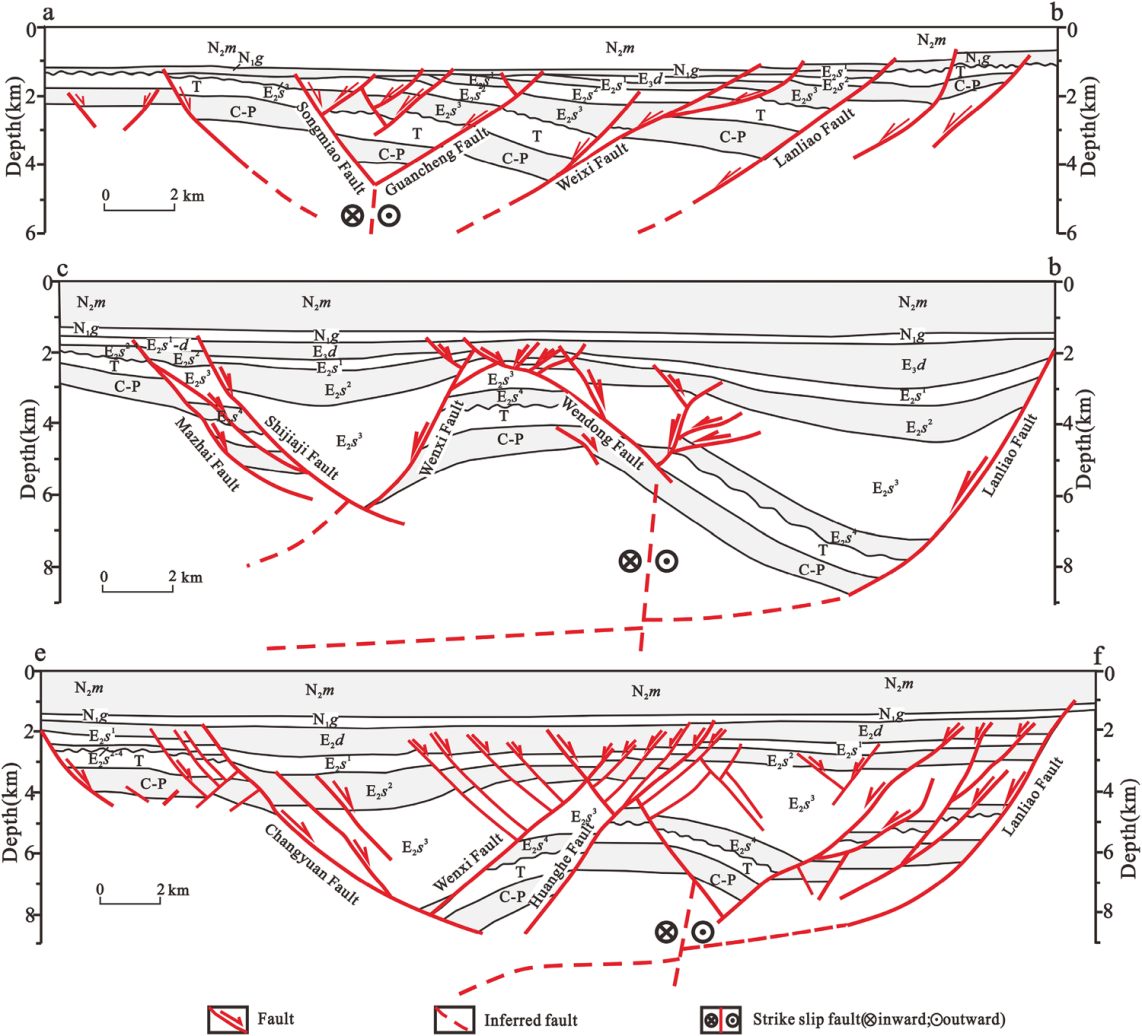
Structural profile of the Dongpu Depression. N_2_m = Pliocene Minghuazhen Formation; N_1_g = Miocene Guantao Formation; E_3_d = Oligocene Dongying Formation; E_2_s = Eocene Shahejie Formation; T = Triassic; C-P = Carboniferous—Permian; O = Ordovician.

The Dongpu Depression is located south of the Bohai Bay Basin with an area of 5300 km^2^ (Fig. 1). This Cenozoic rifted basin was developed on the Paleozoic and Mesozoic basement^19^. The depression includes the western and eastern subdepressions, the Laoliao fault zone, the central uplift zone and the western gentle slope zone (Figs. 1b and 2).

The Dongpu Depression and its surrounding bedrock contain mainly Ordovician limestone and dolomite. The Ordovician in the Dongpu Depression is divided into the Yeli, Liangjiashan, Lower Majiagou, Upper Majiagou and Fengfeng Formations, with thicknesses ranging from 300 to 800 m. The geothermal reservoir types are mainly caves, intercrystalline dissolved pores and fissures. The average porosity and permeability are 2.5% and 7.43 mD, respectively, for the dolomite geothermal reservoirs. The average porosity and permeability are 2.2% and 2.72 mD, respectively, for the limestone geothermal reservoirs^20^. Due to the Huaiyuan and Caledonian-early Hercynian movements, a wide distribution of unconformities developed on the top of the Lower Ordovician Liangjiashan Formation and the Middle Ordovician Fengfeng Formation. In addition, an unconformity was produced between Ordovician and Carboniferous due to the lack of the Upper Ordovician-Devonian strata^20^. In short, the favorable geothermal reservoirs in the Dongpu Depression are concentrated in the Longwangmiao—Maogang—Gaozhuangji—Dongmingji area (type I geothermal reservoirs) and the northwestern Qingfeng-Nanle and southern Machang areas (type II geothermal reservoirs) (Fig. 1b)^20^.

**Figure 3:**
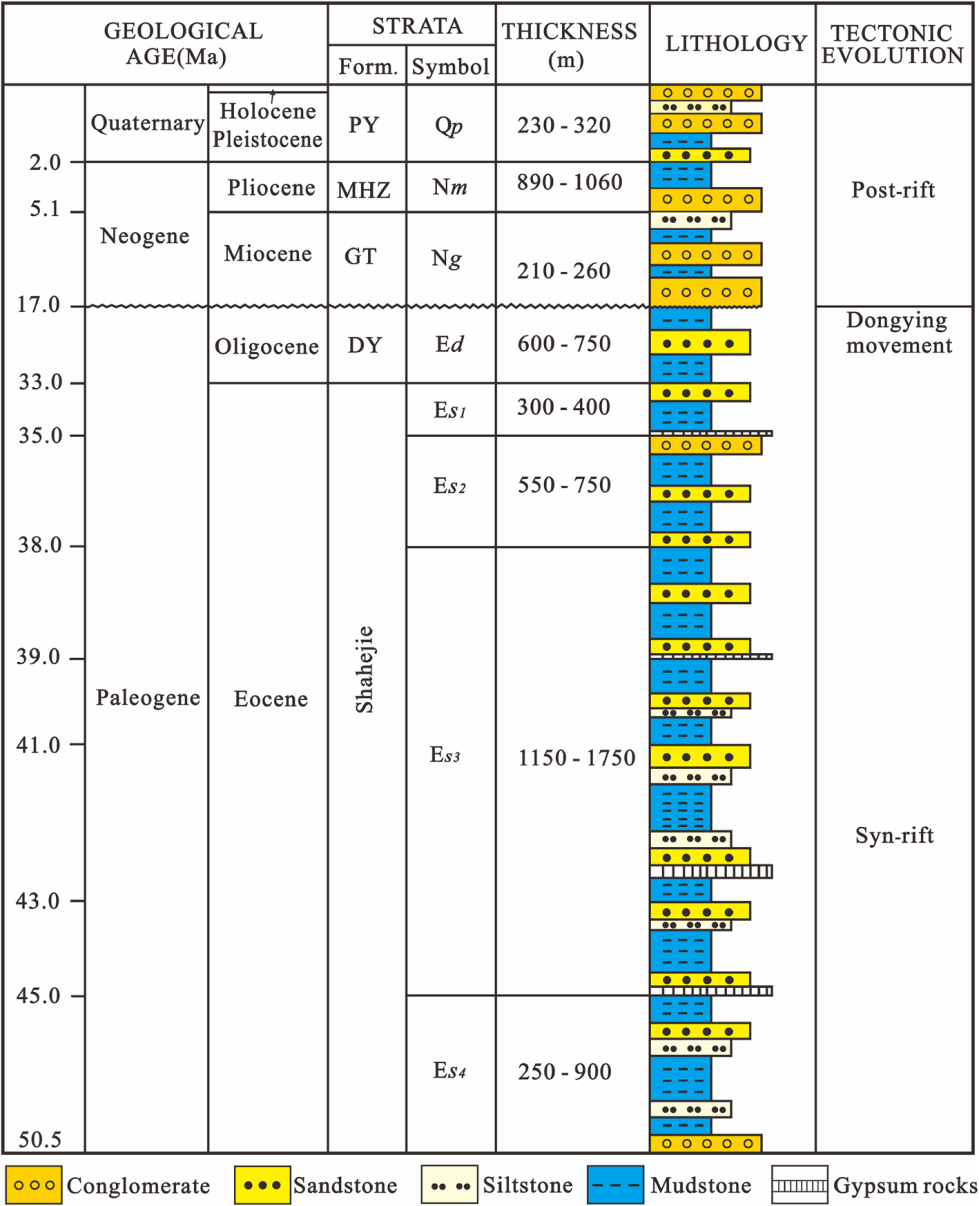
Stratigraphic column of the Dongpu Depression. DY = Dongying Formation; GT = Guantao Formation; MHZ = Minghuazhen Formation; PY = Pingyuan Formation.

**Figure 4:**
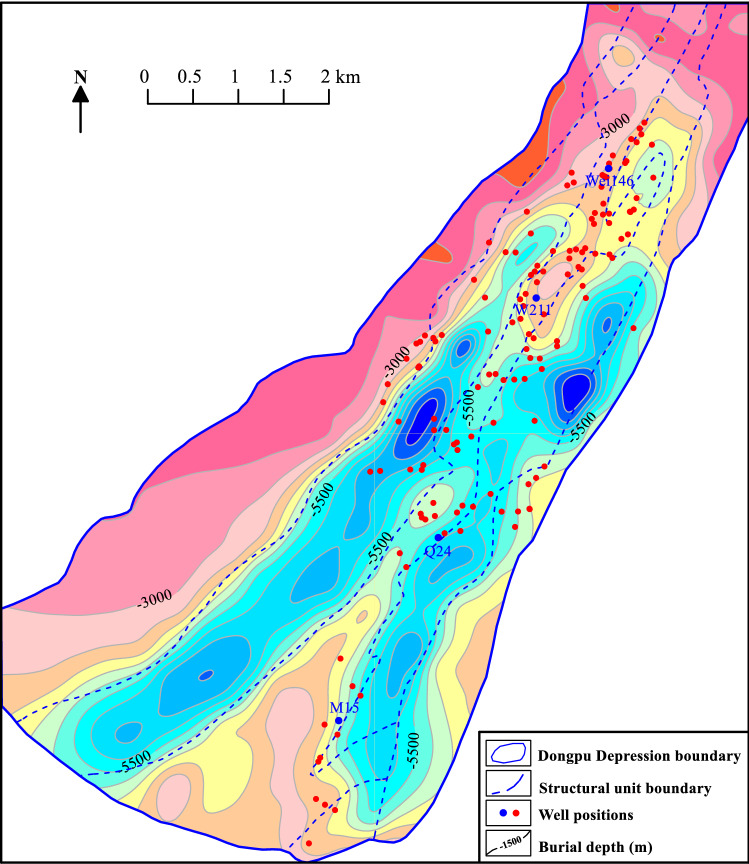
The bottom burial depth of the Cenozoic strata in the Dongpu Depression. Figure was produced by Surfer 15 (https://www.goldensoftware.com/products/surfer/).

The Cenozoic in this area consists of the Shahejie, Dongying, Guantao, Minghuazhen and Pingyuan Formations (Fig. 3). The bottom of the Cenozoic is shallowest in the western slope and northern areas, where is less than 3000 m; it can reach more than 7500 m in the sag area (Fig. 4). The Dongpu Depression is rich in potential water sources for the Ordovician and Cenozoic geothermal reservoirs^14^. The western Changyuan fault and eastern Lanliao fault have water-conducting properties based on the ore-stratification mineralization degree of the Ordovician, so the eastern and western sides of the Dongpu Depression are groundwater source recharge areas. Both the Taihang Mountian and Luxi uplift areas have the large-scale water-collecting capacity, and the Ordovician is exposed in front of the Taihang Mountain and Luxi uplift. The atmospheric precipitation collected in the mountainous areas easily recharges the geothermal field in the Dongpu Depression through Ordovician strata runoff^14^.

The terrestrial heat flow values of the Dongpu Depression range from 37.8 to 106.8 mW/m^2^, with an average value of 66.8 mW/m^2^. The geothermal gradients in the Dongpu Depression range from 20.0 to 56.5 °C/km, with an average value of 34.8 °C/km^21,22^.

## Methodology and parameters

### Methodology

The geothermal resources in the Dongpu Depression are typical thermal conduction types^22^. For each formation, the formation temperature was calculated based on the one-dimensional steady-state thermal conduction equation (Eq. 1)^23^.

1 $$ {\text{T(Z)}} = {\text{T}}_{{0}} + \frac{{{\text{q}}_{{0}} \times {\text{Z}}}}{{\text{K}}} - \frac{{{\text{A}} \times {\text{Z}}^{{2}} }}{{{\text{2K}}}} $$

where T(Z) is the temperature at the calculated depth Z(m), ℃; q_0_ is the surface heat flow at the calculated point, mW/m^2^; K and A are the weighted averages of the thermal conductivity and heat production rate of the stratum rock for the surface to the depth Z(m).

In this paper, firstly, the BasinView software of PRA company was used to establish a three-dimensional geological model, then the different strata temperatures were calculated using the “grid math” function in surfer software based on Eq. (1), and finally, the temperature distribution, geothermal resource type and potential of different geothermal reservoirs were determined.

### Parameters

**Figure 5:**
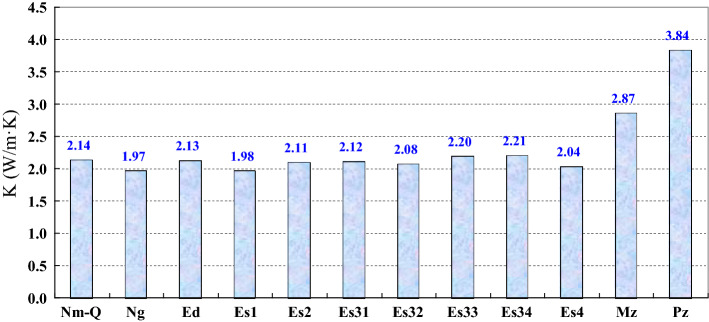
Rock thermal conductivities of different strata. The rock thermal conductivities of the Quaternary, Minghuazhen and Guantao Formations refer to the rock thermal conductivities of the Jiyang Subbasin^24^.

The geological parameters include lithologic data and stratigraphic data. They were obtained from the Zhongyuan Oilfield, SINOPEC. The heat production rate (A) data for the Paleozoic, Mesozoic and Cenozoic refer to data from the adjacent Jiyang Depression, and they are 1.22, 1.51 and 1.43 µW/m^3^, respectively^24^. The rock thermal conductivities are relatively high for the Dongying, Shahejie 2 and 4 Formations (Fig. 5). The surface temperature is set to 15 °C.

## Results

### Temperature distribution for the burial depth

**Figure 6:**
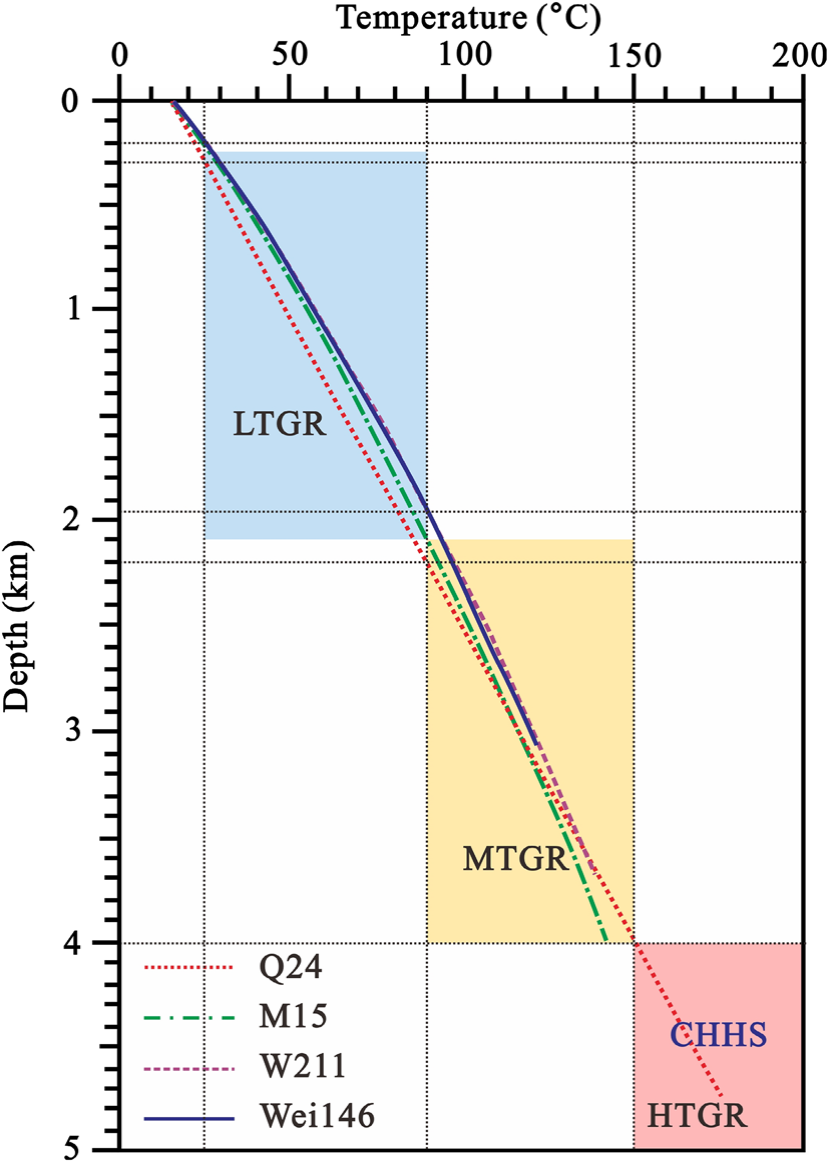
Temperature versus depth for typical wells in the central uplift belt. LTGH = Low-temperature geothermal resources; MTGR = Medium-temperature geothermal resources; HTGR = High-temperature geothermal resources; CHHS = Carbonate-hosted hydrothermal system.

Based on the measured oil testing temperature data^22,25^, the geothermal temperature curve of typical wells in the central uplift belt and the basement temperature of the Dongpu Depression were modeled using the one-dimensional steady-state thermal conduction equation (Eq. 1). According to the classification standard of geothermal resources by temperature^23^, the results show that between 250 and 2100 m depth is a low-temperature geothermal resource zone, with temperatures ranging from 25 to 90 °C; between 2100 and 4000 m depth is a medium-temperature geothermal resource zone, with temperatures ranging from 90 to 150 °C; below 4000 m depth is a high-temperature geothermal resource zone, with temperatures greater than 150 °C (Fig. 6).

### Cenozoic temperature

**Figure 7:**
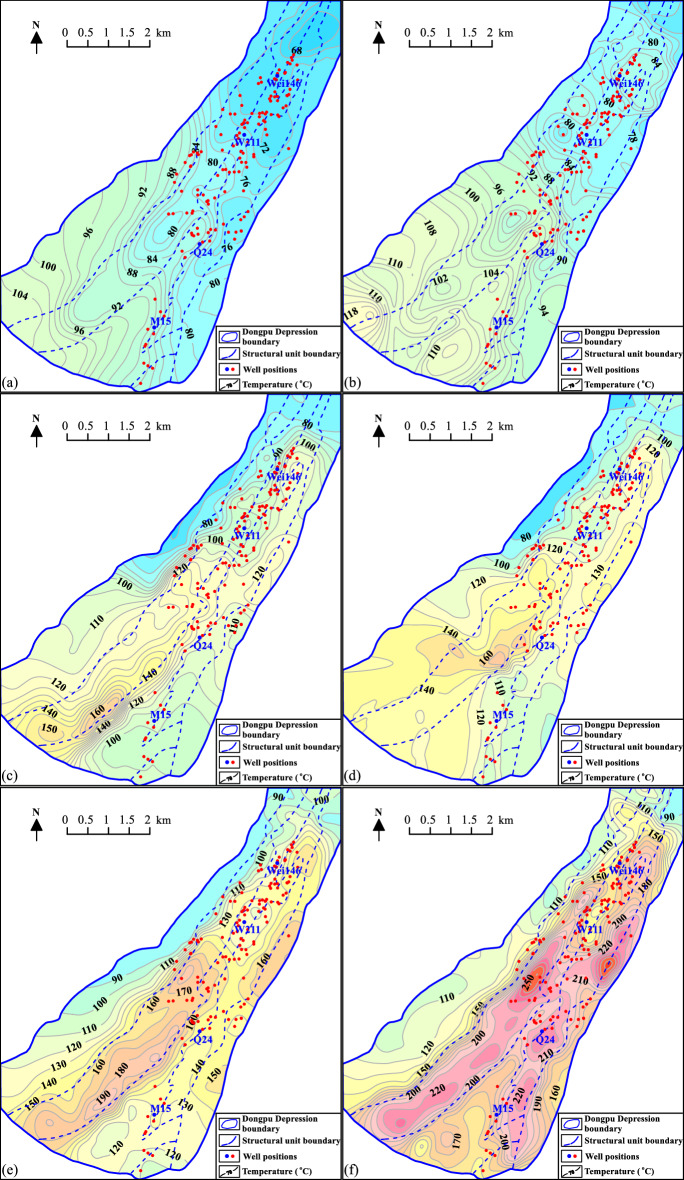
Top surface temperature distribution for the Cenozoic strata in the Dongpu Depression. (**a**) Guantao Formation; (**b**) Dongying Formation; (**c**) Shahejie 1 Formation; (**d**) Shahejie 2 Formation; (**e**) Shahejie 3 Formation; (**f**) Shahejie 4 Formation. Figures were produced by Surfer 15 (https://www.goldensoftware.com/products/surfer/) and BasinView 2012 (https://www.platte.com/software/basinview/).

The top surface temperature of the Guantao Formation is between 63 and 106 °C and is characterized by high temperatures in the southern part of the depression and low temperatures in the northern part of the depression. The temperatures in the eastern subdepression and Lanliao fault zones are low, mainly distributed from 63 to 80 °C (Fig. 7a).

The top surface temperature of the Dongying Formation is between 65 and 120 °C, and its distribution characteristics are similar to those of the top surface temperature of the Guantao Formation (Fig. 7b).

The top surface temperature of the Shahejie 1 Formation increases more than the top surface temperature of the Dongying Formation. The temperature distribution is from 66 to 164 °C and is also characterized by high temperatures in the southern part of the depression and low temperatures in the northern part of the depression. However, the north–south differentiation of temperature is not more evident in the Shahejie 1 Formation than in the Dongying and Guantao Formations, and the high-temperature center of the south sag of the western subdepression and the Qianliyuan sag begins to form (Fig. 7c).

The top surface temperature of the Shahejie 2 Formation is further increased, and its temperature is distributed from 72 to 179 °C. The high-temperature center (greater than 150 °C) gradually shifts from the south to the middle, showing the highest temperature in the south sag of the western subdepression and the Qianliyuan sag. The rest of the depression has lower temperature characteristics (Fig. 7d).

The temperature center of the Shahejie 3 Formation is in the western subdepression, Qianliyuan sag and Pucheng area, and the maximum temperature is more than 190 °C (Fig. 7e).

The top surface temperature of the Shahejie 4 Formation is characterized by a higher temperature in the depression area than in the structural zone. The high-temperature center (greater than 250 °C) is in the middle of the western subdepression and the Qianliyuan sag (Fig. 7f).

### Lower temperature limit of the Ordovician

The basement of the Dongpu Depression includes the Lower Paleozoic marine carbonate rocks and the Upper Paleozoic ocean-continental interface clastic rock strata with a total thickness of nearly 3000 m^20^. The Mesozoic rocks and the Upper Paleozoic Shiqianfeng Formation have basically been denuded in the southern part of the Qiaokou area, and they were in unconformable contact with the overburden^19^. Therefore, the bottom surface temperature of the Cenozoic can be used to represent the lower limit of the Ordovician temperature.

**Figure 8:**
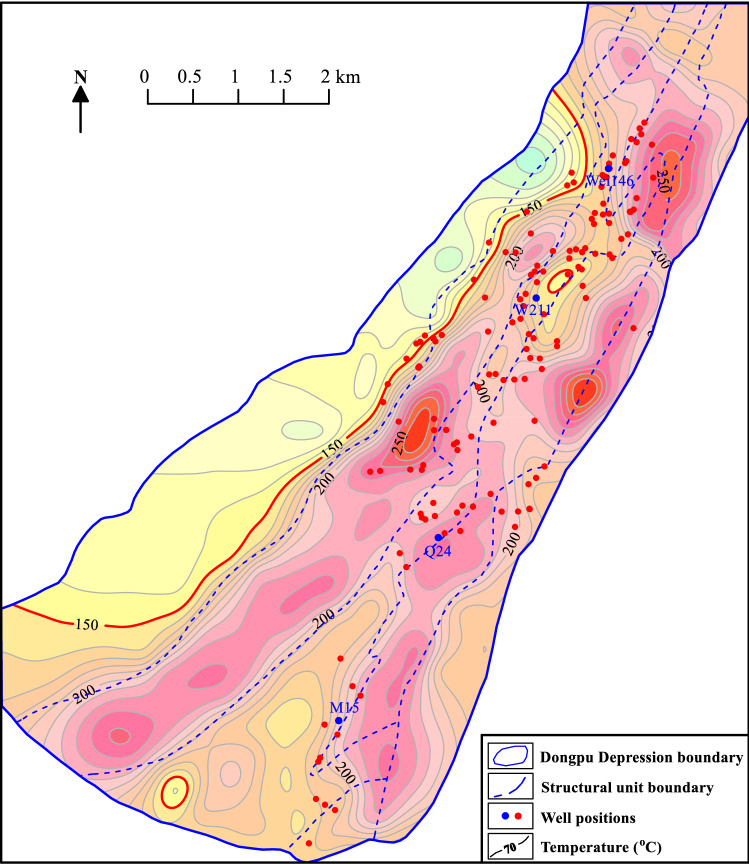
Cenozoic bottom temperature in the Dongpu Depression. Figure was produced by Surfer 15 (https://www.goldensoftware.com/products/ surfer/) and BasinView 2012 (https://www.platte.com/software/basinview/).

The temperature distribution of the bottom surface of the Cenozoic is characterized by higher temperature in the eastern and western depressions, lower temperature in the structural zone, and multiple high-temperature centers. The Cenozoic bottom temperature is more than 150 °C, except in most of the western gentle slope zone. The maximum temperature is distributed in the central part of the western subdepression and the central and northern parts of the eastern subdepression, exceeding 250 °C in these areas (Fig. 8). Therefore, it is speculated that the temperature of the Ordovician rocks in the Dongpu Depression is generally greater than 150 °C, which is favorable for forming high-temperature geothermal resources.

## Discussions

### Method feasibility analysis

As for the oilfields in the middle and late stages of oil and gas exploration, there have been a lot of research results on the water content of potential thermal reservoirs and groundwater systems, which are sufficient to support the geothermal development of oilfields. However, the research on the temperature distributions of the potential thermal reservoirs is relatively weak. Under the precise seismic interpretation, the distributions of the formation thicknesses are relatively accurate. Therefore, based on the three-dimensional geological model, the temperature of the top and bottom surfaces of different potential thermal reservoirs was calculated using the one-dimensional heat conduction equation, which can obtain more accurate temperature distribution characteristics of potential thermal reservoirs, and provide important temperature data for the subsequent calculation of geothermal resources by volume method.

In addition, the accuracy of the calculation results of this method is closely related to the rock thermal parameters (K and A). Therefore, when using this method to calculate the thermal reservoir temperature, it is necessary to test the rock thermal parameters (K and A).

### Geothermal resource classification

The geothermal resources in the Dongpu Depression are heat-conducting geothermal resources^22,25^, which are mainly controlled by a large amount of thermal upwelling from the deep mantle against the background of a relatively thin crust^21^. Therefore, work in the Dongpu Depression should aim to find heat-conducting geothermal resources. The geothermal reservoir temperature is mainly determined by its geothermal gradient, burial depth and rock’s thermal properties^23^. In this paper, the temperatures of different strata are calculated by the one-dimensional steady-state thermal conduction equation. According to the temperature distribution, the geothermal resource types of different strata can be judged. The results can provide valuable temperature information for geothermal resource evaluation and exploration decisions^26,27^.

The geothermal resources include low-temperature, medium-temperature and high-temperature geothermal resources in the Dongpu Depression. The Minghuazhen and Guantao Formations mainly include low-temperature and medium-temperature geothermal resources; the Dongying and Shahejie Formations include low-temperature, medium-temperature and high-temperature geothermal resources; the Ordovician rocks mainly include medium-temperature and high-temperature geothermal resources. Low-temperature geothermal resources are mainly distributed in the Cenozoic deposits in the northern part of the Dongpu Depression and the middle-northern parts of the western slope zone of the Dongpu Depression (Table 1).

**Table 1:** Geothermal resources for the strata in the Dongpu Depression.

Strata	Porosity main distribution (%)	Temperature (°C)				Geothermal resource type
		Top surface		Bottom surface		
		Minimum	Maximum	Minimum	Maximum	
N_2_*m*	20–30	–	–	63	106	Low–medium
N_1_*g*	18–27	63	106	65	120	Low-medium
E_3_*d*	15–25	65	120	66	164	
E_2_*s*^1^	12–20	66	164	67	179	
E_2_*s*^2^	9–15	72	179	69	195	Low–high
E_2_*s*^3^	8–12	78	195	86	263	
E_2_*s*^4^	< 10	86	263	97	270	
Ordovician	3–5	97	270	–	–	Medium–high

### Geothermal resource evaluation

There are abundant geothermal resources in the Dongpu Depression^15^. For sandstone reservoirs below a depth of 3500 m, the compaction is very strong^28^. Therefore, depths less than 3500 m are suggested as the ideal zones for low- and medium-temperature sandstone geothermal resource exploration.

The Minghuazhen, Guantao and Dongying Formations are mostly less than 3500 m deep, with high sandstone contents and high porosities^29^. These strata can form good geothermal reservoirs and are favorable layers for exploring low-temperature and medium-temperature geothermal resources. Although the Shahejie Formation can form low-temperature, medium-temperature and high-temperature geothermal resources, the Shahejie Formation is deeply buried and the geothermal reservoir is relatively poor. The key is to find high-quality geothermal reservoirs. According to the study of sedimentary facies and oil and gas reservoirs^29^, geothermal reservoirs are mainly present in areas where the fan body is developed in the western slope zone and the central uplift, and geothermal resources can be found in these areas. Meanwhile, the Dongpu Depression basement contains a set of Ordovician carbonate strata with secondary porosity, karst caves and dissolution fractures^14,20^, which provide geothermal reservoirs for geothermal resources. The Cenozoic bottom temperature exceeds 150 °C except in most of the western gentle slope zone (Fig. 8). Therefore, the Ordovician carbonate strata were deeply buried, generally more than 4000 m, and had high-temperature geothermal resources.

In addition, for all strata, the overall performance is higher in the southern part of the Dongpu Depression than in the north, and exploration in the southern area can be prioritized during geothermal exploration. In short, the Dongpu Depression has geothermal conditions forming low-temperature, medium-temperature and high-temperature geothermal resources. At the same time, oil and gas exploration has confirmed that the Dongpu Depression has high-quality geothermal reservoirs^29^, and abundant water recharge from the Taihang Mountain and Luxi uplift areas, and deep meteoric water circulation reaching the Ordovician and Cenozoic strata^14^. In the follow-up, we will calculate the geothermal resources of the different thermal reservoirs in the Dongpu depression according to the above results. The potential and all the information collected and described in this paper (porosity, permeability, depth, recharge) will point out the most favorable areas for geothermal exploration and utilization in this area.

## Conclusions

This paper reveals the temperature distribution of different strata, and notes that the Dongpu Depression has low-temperature, medium-temperature and high-temperature geothermal resources. The geological information for exploring and developing the different geothermal resources in the Dongpu Depression are described. The Minghuazhen and Guantao Formations mainly include low-temperature and medium-temperature geothermal resources; the Dongying and Shahejie Formations include low-temperature, medium-temperature and high-temperature geothermal resources; the Ordovician strata mainly include medium-temperature and highest-temperature geothermal resources. In the same formation, the higher temperature geothermal resources are distributed in the southern part of the Dongpu Depression. Therefore, geothermal resource exploration in the Dongpu Depression should prioritize the southern area. In short, Dongpu Depression is rich in geothermal resources, and the research results of this paper provide important information for the next step of ranking the geothermal favorable area and target areas.

## Data Availability

Data are contained in the tables of the article.
